# Switching to ublituximab from prior anti-CD20 monoclonal antibody therapy: a case report series

**DOI:** 10.3389/fimmu.2025.1527102

**Published:** 2025-04-04

**Authors:** Regina Berkovich, Jonathan Calkwood, Heidi Crayton, April Erwin, Simon Faissner, Ralf Gold, Joshua Katz, Mark Leekoff

**Affiliations:** ^1^ Regina Berkovich MD, PhD Inc (MS Center and Research Institute), West Hollywood, CA, United States; ^2^ Department of Neurology, Minnesota Center for Multiple Sclerosis, Plymouth, MN, United States; ^3^ Department of Neurology, MS Center of Greater Washington, Vienna, VA, United States; ^4^ Department of Neurology, Rocky Mountain MS Clinic, Salt Lake City, UT, United States; ^5^ Department of Neurology, Ruhr-University Bochum, St. Josef-Hospital, Bochum, Germany; ^6^ Department of Neurology, The Elliot Lewis Center, Wellesley, MA, United States; ^7^ Atlantic Medical Group Multiple Sclerosis Comprehensive Care Center, Bridgewater, NJ, United States

**Keywords:** anti-CD20, B-cell depletion, disability, magnetic resonance imaging, multiple sclerosis, ocrelizumab, rituximab, ublituximab

## Abstract

**Introduction:**

Anti-CD20 monoclonal antibody (mAb) therapies used to treat multiple sclerosis (MS) differ in their molecular structures, epitope recognition, and mechanisms of CD20-positive (CD20+) cell lysis, which may impact clinical efficacy and tolerability and support within-class switching for patients with suboptimal response to a prior anti-CD20 mAb.

**Patients and methods:**

This is a retrospective case series of 7 individuals with MS treated in private practice or at an MS clinic who switched to ublituximab from a different anti-CD20 mAb therapy due to efficacy or tolerability concerns.

**Case descriptions:**

Details of each case, including clinical and/or radiological outcomes on initial anti-CD20 mAb therapy, reasons for switching, and outcomes after starting ublituximab therapy are provided.

**Discussion:**

These cases highlight suboptimal B-cell depletion, inadequate MS disease control, and/or tolerability concerns in people with MS who had clinical improvements or stabilization of disease following a switch from ocrelizumab or rituximab to ublituximab.

**Conclusion:**

Within-class switching from a prior anti-CD20 mAb therapy to ublituximab is feasible and may improve outcomes in some people with MS.

## 1 Introduction

Ocrelizumab, ofatumumab, and ublituximab are approved anti-CD20 monoclonal antibody (mAb) therapies for multiple sclerosis (MS), and rituximab has been utilized off label (1–7). Efficacy of these 4 mAb therapies results from depletion of CD20-positive (CD20+) B cells and possibly of CD20+ T cells (8, 9). Complement-dependent cytotoxicity (CDC) is the dominant mechanism of CD20+ B-cell lysis by rituximab and ofatumumab, whereas antibody-dependent cellular cytotoxicity (ADCC) predominates with ocrelizumab and ublituximab (10). ADCC can be enhanced by glycoengineering of the fragment crystallizable (Fc) region, which allows for closer interaction with and increased affinity for Fcγ receptor IIIa (FcγRIIIa) on natural killer cells (11–13). Glycoengineering can also overcome effects of FcγRIIIa polymorphisms (14), which have been associated with variable clinical responses to rituximab (15–17). Ublituximab has a glycoengineered Fc region and the highest binding and relative affinity for FcγRIIIa 158V and FcγRIIIa 158F variant receptors compared with other anti-CD20 mAb therapies, and it has the highest *in vitro* ADCC activity (18–20).

Differences in structure and function across anti-CD20 mAbs raises the question as to whether patients who experience suboptimal response to one anti-CD20 mAb may benefit from within-class switching. Here we present 7 cases of individuals with MS who switched to ublituximab from a prior anti-CD20 mAb therapy due to efficacy and/or tolerability concerns.

Note: All infusions and symptomatic medications were dosed consistent with U.S. Food and Drug Administration prescribing information or European Medicines Agency summary of product characteristics labeling, unless otherwise described. No adverse events were noted other than those described.

## 2 Case descriptions

### 2.1 Case 1

A 25-year-old Latina female with left optic neuritis presented to the clinic for her initial evaluation in August 2014. Brain and cervical spine magnetic resonance imaging (MRI) showed T2-enhancing lesions in the brain and thoracic spinal cord. She was diagnosed with relapsing MS and began treatment with fingolimod in September 2014. In March 2015, she experienced numbness in her left thigh, and a brain MRI showed a new enhancing right periventricular lesion. She had not been taking fingolimod consistently but started taking it regularly and remained stable for the next 4 years with minimal baseline symptoms (Expanded Disability Status Scale [EDSS] score 1.5). A routine follow-up MRI in May 2019 showed multiple enhancing cerebral lesions, although she reported no symptoms.

**Figure 1:**
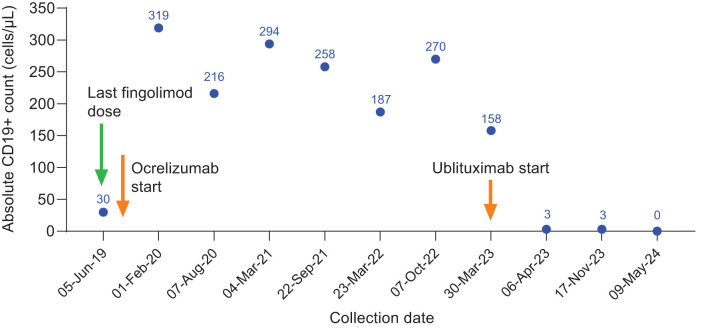
CD19+ counts in Case 1 during ocrelizumab treatment and after switch to ublituximab treatment. Ocrelizumab treatment was initiated in July 2019, with last ocrelizumab dose in October 2022. Ublituximab treatment was started in March 2023, with robust B-cell depletion (3 cells/μL) observed at 1 week after first ublituximab dose.

**Figure 2:**
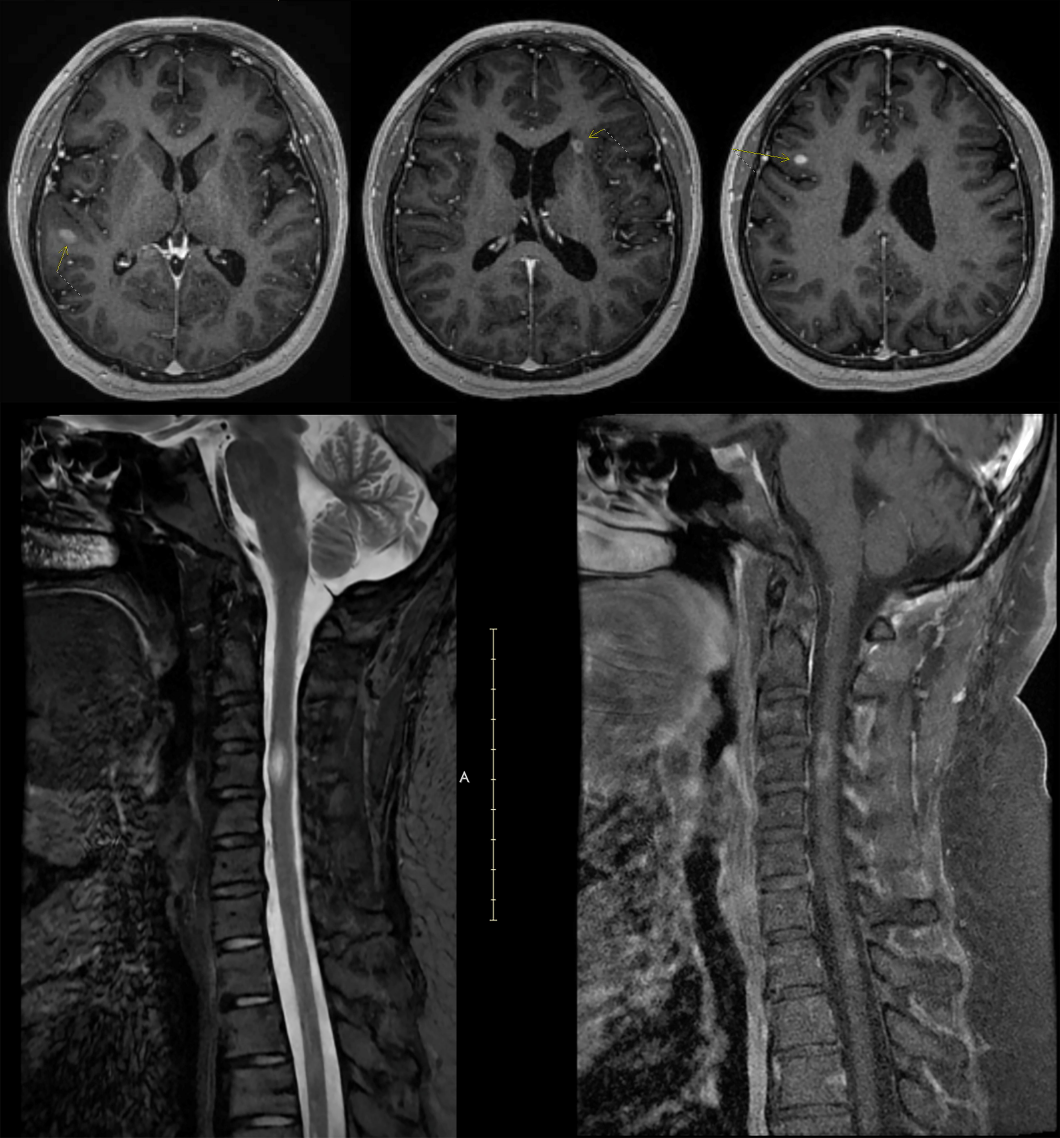
Radiographic images of Case 1 obtained after initiation of ocrelizumab. Cervical MRI obtained 5 months after the seventh cycle of ocrelizumab showing a new gadolinium-enhancing lesion in the cervical cord at C4. MRI, magnetic resonance imaging.

She began treatment with ocrelizumab and had her first split dose in July 2019, 4 weeks after stopping fingolimod. Over the next 3.5 years, she received 7 cycles of ocrelizumab and remained clinically stable with no new MRI lesions. B-cell count was checked prior to each 6-month ocrelizumab infusion, but it was always in the normal range, indicating suboptimal B-cell depletion (**Figure 1**). Five months after her seventh cycle of ocrelizumab, she began having numbness and paresthesia in her left leg ascending to the level of her chest and left leg weakness. An MRI showed a new gadolinium-enhancing lesion in the cervical cord at C4 (**Figure 2**).

Because she had clinical and radiologic breakthrough and normal CD19 counts at the time of her last ocrelizumab infusion, she was switched from ocrelizumab to ublituximab in March 2023, receiving a single 450-mg dose 6 months after her last ocrelizumab infusion (October 2022). B-cell subsets were checked 1 week after receiving ublituximab, and the absolute B-cell count was 3 cells/μL (0% B cells). Six months later, her left leg numbness and heaviness had resolved, and the only abnormalities on neurological exam were mildly reduced vibration sense and hyperreflexia in the left leg (EDSS score 1.5). Over the following 12 months, she had 2 additional cycles of ublituximab and no further clinical episodes or new MRI lesions. CD19+ B-cell counts at each 6-month ublituximab dose showed full B-cell depletion (**Figure 1**).

### 2.2 Case 2

A 53-year-old White female presented to the clinic for evaluation in May 2014. Her initial onset of disease occurred in 2012 when she experienced acute optic neuritis with an enhancing MRI lesion in the right optic nerve. In November 2016, she had an episode of new paresthesia in the right upper and lower extremities. A neurological exam and MRI of the brain and spinal cord were normal at that time and remained unchanged until August 2018 when she developed right upper extremity numbness. An MRI of the cervical spine in October 2018 showed a contrast-enhancing lesion, and a subsequent brain MRI identified new lesions in the posterior fossa, corpus callosum, and subcortical white matter, with 2 contrast enhancing lesions. Five unique oligoclonal bands and an elevated immunoglobulin G (IgG) index were found in the cerebrospinal fluid (CSF), and tests for antibodies to aquaporin-4 and glutamic acid decarboxylase were negative.

**Figure 3:**
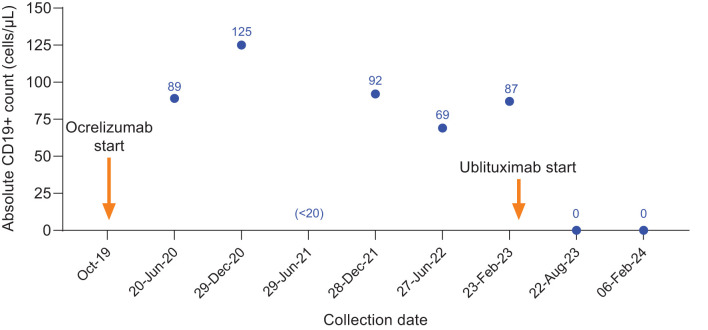
CD19+ counts in Case 2 during ocrelizumab treatment and after switch to ublituximab treatment. Ocrelizumab treatment was initiated in October 2019, with last ocrelizumab dose received in June 2022. The individual skipped the December 2022 ocrelizumab infusion with the intent to switch to ublituximab and initiated ublituximab treatment in March 2023, with complete B-cell depletion observed at the subsequent preinfusion blood collections.

In February 2019, she started treatment with glatiramer acetate for relapsing MS, but experienced a significant brainstem relapse after 1 month and required plasmapheresis treatment. She was advised that she had highly active disease and would benefit from a more efficacious disease-modifying therapy (DMT) for MS; treatment with ocrelizumab was initiated in October 2019. With the first 3 administrations of ocrelizumab, she experienced initial infusion reactions of throat swelling and irritation, itching in the ears, and nausea; her experiences of throat and ear irritation continued and required infusion rate interruption and additional antihistamine treatment at each administration. In April 2020, while receiving treatment with ocrelizumab, new asymptomatic nonenhancing MRI lesions were identified on her brain MRI. In July 2020, 8 months following ocrelizumab initiation, a brainstem sensory relapse occurred. This relapse responded well to steroids, and her EDSS score recovered to her pretreatment baseline of 4.0. During the remainder of her course of ocrelizumab, her EDSS score remained stable at 4.0, and no new or enhancing MRI lesions or relapses were observed. She maintained a consistent infusion frequency until the end of 2022; however, absolute CD19+ cell counts taken prior to each 6-month ocrelizumab infusion indicated insufficient B-cell depletion or early repopulation, with CD19+ cell counts as high as 125 cells/μL (**Figure 3**).

Due to concerns of early persistent CD19+ cell repletion, MS disease activity, and repeated infusion-related reactions, treatment with ocrelizumab was discontinued and ublituximab was initiated in March 2023. The initial doses of ublituximab were well tolerated without complications, but her August 2023 ublituximab infusion was temporarily interrupted due to itching in the ears and mouth. After administering IV diphenhydramine 25 mg, the infusion was restarted approximately 30 minutes later and completed without any other complications. The most recent infusion (February 2024) was completed without interruption and was well tolerated. She subjectively assessed the infusion-related symptoms with ublituximab as less severe than those with ocrelizumab. Absolute CD19+ cell counts taken prior to each 6-month ublituximab infusion in August 2023 and February 2024 showed full B-cell depletion (0 cells/μL; **Figure 3**), her clinical course on ublituximab based on approximately 18 months post-treatment follow-up was free of new symptoms or MRI changes, and EDSS score remained 4.0.

### 2.3 Case 3

A 54-year-old White female presented to the clinic for her initial evaluation in April 2022 with symptoms of paresthesia, coordination difficulty, and weakness of the right hand for the past 3 years; she also reported progressive weakness in the left lower extremity in the last 6 months and decreased endurance, leading her to apply for short-term disability. She was diagnosed with active secondary progressive MS based on brain MRI showing supratentorial and infratentorial white matter lesions with minimal enhancement as well as cervical and thoracic spine lesions, spinal tap showing 3 unique oligoclonal bands, and neurological examination findings. Her symptoms included spasticity, stiffness upon waking, heat intolerance, and suspected cognitive issues as well as difficulty walking long distances but no recent falls.

Ocrelizumab treatment and baclofen for spasticity were initiated in May 2022. In November 2022, at the time of her third ocrelizumab infusion, her B-cell count was 182 cells/μL, indicating early B-cell repopulation or incomplete depletion; thus, an earlier ocrelizumab infusion was scheduled. In March 2023 (4 months post infusion), her B-cell count was 42 cells/μL, and she noted that her condition was progressively worsening and felt like ocrelizumab was “wearing off.”

Due to early B-cell repopulation and a feeling of treatment “wearing off,” she transitioned to ublituximab, receiving her first infusion in May 2023. Dalfampridine treatment was also initiated at this time. At the September 2023 appointment, her B-cell count was 0 cells/μL and she did not report feeling a “wearing off” of ublituximab. She received her next ublituximab infusion in October 2023 and did not report any significant change or complaints in December 2023. At the end of March 2024, she again denied a “wearing off” effect of ublituximab, and her B-cell count on March 27, 2024, was 2 cells/μL (prior to the planned infusion in April 2024).

### 2.4 Case 4

A 38-year-old Black female presented to the clinic for her initial evaluation in July 2020. She was diagnosed with relapsing MS in 2015, presenting with symptoms of decreased hand dexterity and a “band like” sensation around her waist. Brain and cervical spinal cord MRI showed signal changes consistent with demyelination, and she began treatment with glatiramer acetate. She and her healthcare provider at the time decided to switch from glatiramer acetate to ocrelizumab due to injection reactions and injection fatigue.

**Figure 4:**
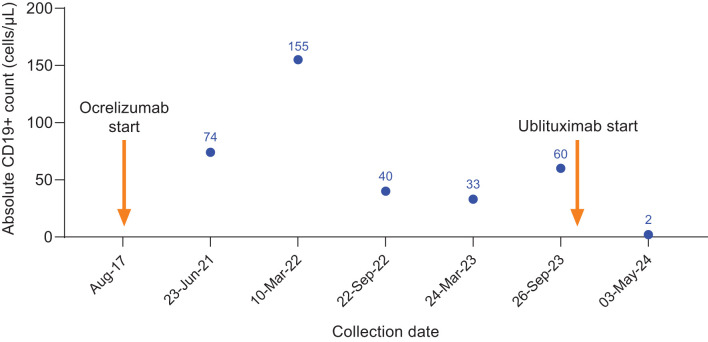
CD19+ counts in Case 4 during ocrelizumab treatment and after switch to ublituximab treatment. Ocrelizumab treatment was initiated in August 2017, with last ocrelizumab dose in March 2023. Ublituximab treatment was started in October 2023, with complete B-cell depletion observed at the subsequent preinfusion blood collection.

She initiated ocrelizumab treatment in August 2017, receiving regular infusions until her last dose of ocrelizumab in March 2023. Her absolute CD19+ counts during ocrelizumab treatment indicated insufficient B-cell depletion (**Figure 4**). Although MRI findings were stable, she reported bladder urgency, bowel issues, and fatigue.

Due to ongoing symptoms, insufficient B-cell depletion, and a sense of “wearing off” with ocrelizumab, the decision was made to switch to ublituximab. In September 2023, 7 months after her last ocrelizumab dose and prior to her initial ublituximab dose, laboratory evaluation revealed an absolute CD19+ count of 60 cells/μL. The patient initiated ublituximab and received her first and second infusions in October 2023. Prior to her subsequent dose in May 2024, her CD19+ count was 2 cells/μL. Since the switch to ublituximab, her fatigue has been more manageable, her bowel function has normalized, and she has less bladder urgency.

### 2.5 Case 5

A 36-year-old White male presented for his initial evaluation in November 2015. He was diagnosed with relapsing MS at 18 years of age, with a first manifestation of optic neuritis, and was treated with multiple platform therapies over the course of 8.5 years. Over the first 4 years he was treated with 22-µg subcutaneous interferon β-1a and experienced several relapses without substantial residual disability until a severe relapse that, despite adequate treatment, necessitated a bilateral walking aid. Treatment was switched in 2014 to teriflunomide for 1.5 years, but his disability continued to worsen. After another relapse with ataxia and chanting speech, treatment was escalated to fingolimod (August 2015) for 4 months. During an event of acute urinary retention, he was first admitted to a specialized MS center with an EDSS score of 8.0, marked ataxia, and inability to stand without help. Treatment was switched (December 2015) to rituximab 500 mg every 6 months and continued for 4.5 years. During this time, his EDSS score improved to 7.5, but therapy was later switched to ocrelizumab due to an impression of “wearing off” of rituximab.

Following the first infusion of ocrelizumab in February 2020, he mentioned a deterioration of the ataxia and complained about fatigue, cephalgia, and dysesthesia, all considered most likely due to an infusion reaction, and he refrained from continuing ocrelizumab. He received an implantation of a deep brain stimulation system (Boston Scientific, Marlborough, MA), resulting in marked improvement of resting tremor, intention tremor, and ataxia; a baclofen pump was also implanted to treat severe spasticity. Immunotherapy was switched again back to rituximab for another 3.5 years.

Due to loss of insurance coverage for rituximab for off-label use, he was switched to ublituximab in February 2024. The first treatment cycle was well tolerated, and his leg strength improved gradually within the following weeks. Notably, he was able to walk again using a walking frame with the help of a physiotherapist for the first time in 6 years, and his EDSS score improved from 8.0 to 7.5.

### 2.6 Case 6

A 43-year-old Hispanic female presented for her initial evaluation in November 2018. She was initially diagnosed with relapsing progressive MS in 2008 and with comorbid rheumatoid arthritis (RA) in 2010. At the time of her MS diagnosis, an MRI of the brain, cervical, and thoracic spine revealed multiple lesions, and her CSF was positive for oligoclonal bands. She was initially treated in 2010 with IV rituximab, starting with two 1000-mg infusions separated by 2 weeks then 1000 mg every 6 months, prescribed by her rheumatologist for anticipated treatment of both RA and MS. During an 18-month period from 2021 to 2022 while on rituximab, she experienced several MS relapses, leading to accumulation of disability and an EDSS score change from 5.0 to 6.0 and, shortly thereafter in October 2022, to a score of 6.5. It was concluded that although her RA management was satisfactory, her MS treatment was inadequate.

Due to inadequate control of MS, rituximab was discontinued and ublituximab was initiated, with the rationale that, since both drugs are in the same pharmacological class, ublituximab being an approved DMT for MS may provide improved MS disease control and be adequate for RA. She received her first ublituximab infusion in January 2023, and in a subsequent follow-up in July 2023, her IgG profile was within normal limits and CD19+ counts indicated full B-cell depletion. An MRI from July 2023 revealed multiple demyelinating, brain, brainstem, and spinal cord lesions consistent with MS but no new or active lesions compared with 2022 MRI findings. As of May 2024, she had received 4 ublituximab infusions per prescribing information; all were well tolerated and completed within 1 hour, and no adverse events were noted. She reported feeling stable, and no MS relapses or further EDSS progression have been reported since starting ublituximab. Incidentally, no ill effects were observed in her comorbid condition of interest, rheumatoid arthritis.

### 2.7 Case 7

A 33-year-old White female presented for her initial evaluation to the clinic in June 2021 with a history of MS diagnosed in December 2017, when she was 7 months postpartum. At the time of diagnosis, she experienced a loss of sensation in her left arm and began dropping things. Brain MRI showed nonenhancing demyelinating lesions, and cervical spine MRI showed an expansile enhancing lesion at the C4-C5 level. CSF was positive for white blood cells (39 cells/μL) and oligoclonal bands (11 unique bands) and showed an elevated IgG index of 2.07. She was started on glatiramer acetate but discontinued due to side effects, subsequently experiencing relapses in 2018, 2019, and 2021. An MRI in March 2021 revealed significant areas of disease activity in the brain and cervical and thoracic spine.

In June 2021, a physical exam showed left pronator drift, left leg weakness, impaired tandem walking, and ≥4 ankle jerks with unsustained clonus. She initiated ocrelizumab in June 2021 and maintained the standard 6-month infusion frequency. Brain MRI in December 2022 showed stable white matter disease with no abnormal enhancement. Ocrelizumab fast infusion protocol was initiated in January 2023. In July 2023, she reported tachycardia that had persisted after the January ocrelizumab infusion, but she proceeded with her scheduled July 2023 infusion. Brain MRI in November 2023 showed multiple scattered areas of increased T2 and fluid-attenuated inversion recovery signal within the periventricular, deep, and subcortical white matter. One of the lesions adjacent to the posterior horn of the right lateral ventricle demonstrated subtle enhancement consistent with active demyelination.

Due to the enhancing lesion, the decision was made to switch from ocrelizumab to ublituximab. Initial doses of ublituximab were completed in January 2024. Subsequently, the patient reported improved headaches, complete resolution of dizziness, and no new or worsened symptoms at the March 2024 follow-up visit. Brain MRI in April 2024 showed stable white matter disease with no new or enhancing lesions identified. Cervical and thoracic spine MRI showed multifocal, short-segment T2 hyperintense lesions throughout the cervical and thoracic spinal cord, improved in size and conspicuity compared with the March 2021 findings, with no abnormal enhancement.

## 3 Discussion

Not all patients respond optimally to anti-CD20 mAb therapy, and suboptimal B-cell depletion and breakthrough MS disease activity have been reported. In a large real-world study, B-cell repletion (defined as ≥1% of lymphocyte count) at 6 months occurred in 26% of people treated with ocrelizumab and was associated with higher MRI activity (21). In the pivotal ocrelizumab studies, body weight impacted ocrelizumab exposure and depletion of B cells, and >30% of participants in the lowest exposure quartile had incomplete B-cell depletion at week 96 (22). Some small studies have suggested that anti-CD20 mAb efficacy (23, 24) and safety (24) could vary depending on race, but results are far from definitive. Furthermore, a “wearing off” effect with anti-CD20 mAbs is commonly reported, occurring in up to 61% of individuals, with fatigue, cognitive symptoms, mobility issues, and sensory symptoms reported most frequently, though notably wearing off symptoms have not been found to correlate with B-cell return, and the pathophysiology is unclear (25–28). In the present case series, suboptimal responses with anti-CD20 mAb therapy included issues with tolerability, B-cell depletion control, and objective disease breakthrough. While it would be logical to consider switching to a DMT with a different mechanism of action, there could be various reasons to advocate for remaining within the class; for example, to name just one, the presence of a comorbid (to MS), purely B-cell driven condition such as RA, as was presented in Case 6. The authors feel that it is of interest to share these experiences exactly due to the common belief that switching within pharmacological class may not be a well-justified treatment plan. However, newer pharmacological technologies may assist in overcoming previously observed mechanisms behind suboptimal drug effects, thus providing theoretical basis for the above clinical exploration.

Multiple review articles have elucidated the differentiating features of the available anti-CD20 mAbs used in relapsing MS, including their respective epitopes, mechanisms of B-cell depletion (CDC and ADCC), and clinical profiles (efficacy and safety) (8, 10, 19, 29). Therefore, it is rational to suggest suboptimal response to one anti-CD20 mAb does not preclude improved response on a different anti-CD20 mAb, supporting in-class treatment switching.

This case series explores experiences of people with MS for whom a switch within the class was performed; our findings suggest that suboptimal response to one anti-CD20 mAb does not necessarily portend suboptimal response to a different anti-CD20 mAb. This case series presents preliminary clinical evidence on an emerging treatment decision that neurologists and patients may consider and that may present another option in addition to switching to a DMT of alternative class.

In several cases in our series, following suboptimal B-cell depletion with other anti-CD20 mAb therapies, robust B-cell depletion was observed after the initial dose of ublituximab. This could be due to the enhanced affinity for all FcγRIIIA variants and stronger ADCC with ublituximab versus other anti-CD20 mAb therapies as a result of its glycoengineered Fc region (18–20). Several individuals had improvement in their MS disease activity and/or better tolerability upon switching to ublituximab from rituximab or ocrelizumab, and individuals did not report a “wearing off” effect with ublituximab.

Limitations of this case series include that this was not a prospective study but rather a compilation of individual cases with relatively short follow-up.

## 4 Conclusion

This case series highlights that anti-CD20 mAb therapies are not interchangeable in terms of clinical profile at the individual patient level. People with MS experiencing clinical efficacy and/or tolerability concerns with rituximab or ocrelizumab can be successfully switched to ublituximab.

## Data Availability

The raw data supporting the conclusions of this article will be made available by the authors, without undue reservation.
