# Corrigendum: Switching to ublituximab from prior anti-CD20 monoclonal antibody therapy: a case report series

**DOI:** 10.3389/fimmu.2025.1609054

**Published:** 2025-06-13

**Authors:** Regina Berkovich, Jonathan Calkwood, Heidi Crayton, April Erwin, Simon Faissner, Ralf Gold, Joshua Katz, Mark Leekoff

**Affiliations:** ^1^ Regina Berkovich MD, PhD Inc (MS Center and Research Institute), West Hollywood, CA, United States; ^2^ Department of Neurology, Minnesota Center for Multiple Sclerosis, Plymouth, MN, United States; ^3^ Department of Neurology, MS Center of Greater Washington, Vienna, VA, United States; ^4^ Department of Neurology, Rocky Mountain MS Clinic, Salt Lake City, UT, United States; ^5^ Department of Neurology, Ruhr-University Bochum, St. Josef-Hospital, Bochum, Germany; ^6^ Department of Neurology, The Elliot Lewis Center, Wellesley, MA, United States; ^7^ Atlantic Medical Group Multiple Sclerosis Comprehensive Care Center, Bridgewater, NJ, United States

**Keywords:** anti-CD20, B-cell depletion, disability, magnetic resonance imaging, multiple sclerosis, ocrelizumab, rituximab, ublituximab

In the published article, there was an error in affiliation 1. Instead of “Berkovich MS Center and Research Institute, West Hollywood, CA, United States,” it should be “Regina Berkovich MD, PhD Inc (MS Center and Research Institute), West Hollywood, CA, United States.”

Also, there was an error in **Figure 1**, **Figure 3**, and **Figure 4** as published. The y-axis titles of these figures contained a typo listing the unit of measure as “cells/¼ L”; the correct unit of measure is “cells/µL.” The corrected **Figure 1**, **Figure 3**, and **Figure 4** and their captions appear below.

**Figure 1:**
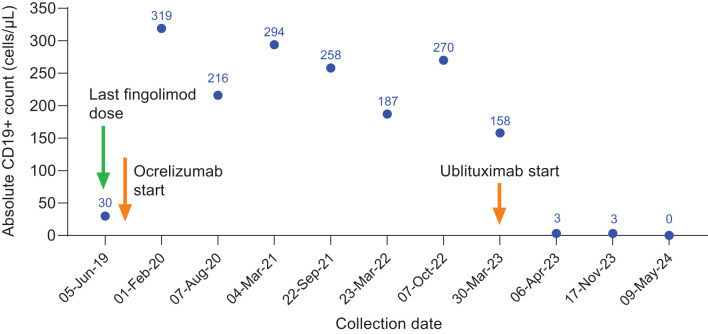
CD19+ counts in Case 1 during ocrelizumab treatment and after switch to ublituximab treatment. Ocrelizumab treatment was initiated in July 2019, with last ocrelizumab dose in October 2022. Ublituximab treatment was started in March 2023, with robust B-cell depletion (3 cells/μL) observed at 1 week after first ublituximab dose.

**Figure 3:**
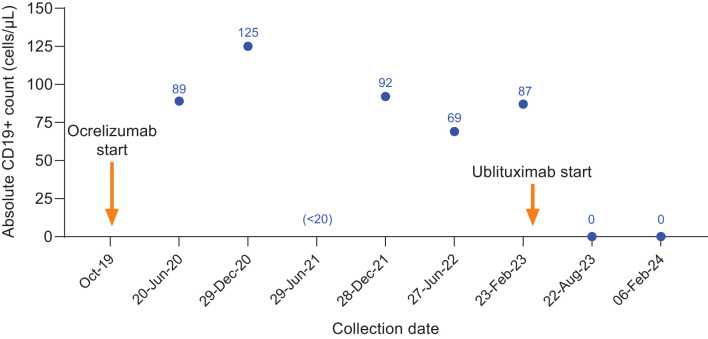
CD19+ counts in Case 2 during ocrelizumab treatment and after switch to ublituximab treatment. Ocrelizumab treatment was initiated in October 2019, with last ocrelizumab dose received in June 2022. The individual skipped the December 2022 ocrelizumab infusion with the intent to switch to ublituximab and initiated ublituximab treatment in March 2023, with complete B-cell depletion observed at the subsequent preinfusion blood collections.

**Figure 4:**
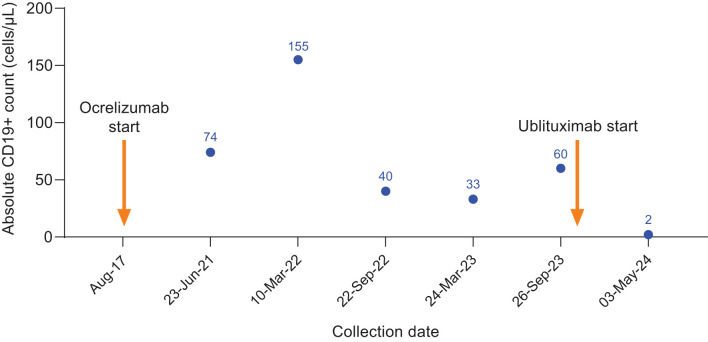
CD19+ counts in Case 4 during ocrelizumab treatment and after switch to ublituximab treatment. Ocrelizumab treatment was initiated in August 2017, with last ocrelizumab dose in March 2023. Ublituximab treatment was started in October 2023, with complete B-cell depletion observed at the subsequent preinfusion blood collection.

The authors apologize for this error and state that this does not change the scientific conclusions of the article in any way. The original article has been updated.

